# Familiarization Protocol Influences Reproducibility of 20-km Cycling Time-Trial Performance in Novice Participants

**DOI:** 10.3389/fphys.2017.00488

**Published:** 2017-07-20

**Authors:** Andrew W. Hibbert, François Billaut, Matthew C. Varley, Remco C. J. Polman

**Affiliations:** ^1^Institute of Sport, Exercise and Active Living, Victoria University Melbourne, VIC, Australia; ^2^College of Sport and Exercise Science, Victoria University Melbourne, VIC, Australia; ^3^Department of Kinesiology, Université Laval Quebec, QC, Canada; ^4^School of Exercise and Nutrition Sciences, Queensland University of Technology Brisbane, QLD, Australia

**Keywords:** familiarization, exercise research design, pacing, performance, time-trial

## Abstract

**Introduction:** Exercise performance is reproducible in experienced athletes; however, less trained participants exhibit greater variability in performance and pacing. To reduce variability, it is common practice to complete a familiarization prior to experimental testing. However, there are no clear guidelines for familiarizing novice participants to a cycling time-trial (TT), and research findings from novice populations may still be influenced by learning effects. Accordingly, the aims of this study were to establish the variability between TTs after administering differing familiarization protocols (duration or type) and to establish the number of familiarization trials required to limit variability over multiple trials.

**Methods:** Thirty recreationally active participants, with no prior experience of a TT, performed a 20-km cycling TT on five separate occasions, after completing either a *full* (FF, 20-km TT, *n* = 10), a *half* (HF, 10-km TT, *n* = 10) or an *equipment* familiarization (EF, 5-min cycling, *n* = 10).

**Results:** Variability of TT duration across five TTs was the lowest after completing FF (*P* = 0.69, η_*p*_^2^ = 0.05) compared to HF (*P* = 0.08, η_*p*_^2^ = 0.26) and EF (*P* = 0.07, η_*p*_^2^ = 0.21). In the FF group after TT2, the effect size for changes in TT duration was small (*d* < 0.49). There were large differences between later TTs in HF (*d* = 1.02, TT3-TT4) and EF (*d* = 1.12, TT4-TT5). The variability in mean power output profiles between trials was lowest within FF, with a similar pacing profile reproduced between TT3-TT5.

**Discussion:** Familiarization of the exercise protocol influenced reproducibility of pacing and performance over multiple, maximal TTs, with best results obtained after a full experience of the exercise compared to HF and EF. The difference of TT1 to later TTs indicates that one familiarization is not adequate in reducing the variability of performance for novice participants. After the FF and an additional TT, performance changes between TTs were small, however, a reproducible pacing profile was not developed until after the FF and two additional TTs. These findings indicate that a minimum of three full familiarizations are necessary for novice participants to limit systematic error before experimental testing.

## Introduction

During exercise testing, particular care is needed to ensure reliability from testing procedures, equipment, and the “internal” ability of participants to achieve the goals of the task (Hopkins, 2000). As such, when planning a repeated-measure design, within-subject variability must be considered. This is especially the case for closed-loop exercise tests allowing for continuous adjustments in pace that may impact overall performance. This may be a concern for self-paced exercises, such as a time-trial (TT), as intensity varies when attempting to complete the distance as quickly as possible. Consequently, repeating tests may result in different pacing strategies and, therefore, performance based on the preceding familiarization.

Previous studies have shown that cycling TT duration and mean power are relatively reproducible in trained cyclists (Sporer and McKenzie, 2007; Zavorsky et al., 2007). This comes as no surprise as athletes who are familiar with this exercise outside of laboratory conditions are likely to have deep-rooted pacing strategies that match the requirements of the given exercise (Mauger et al., 2010; Thomas et al., 2012). However, well-trained athletes are not immune to variations in performance. During multiple 4-km (Ansley et al., 2004) and 20-km TTs (Thomas et al., 2012), competitive cyclists have shown an indication for an increased starting power output in the first TT, that is progressively reduced over two repeated trials. This finding is also true for novice participants performing three 2-km TTs (Corbett et al., 2009). Conversely, novices have also produced a greater mean power output in the third of three 10-km TTs (Foster et al., 2009). Within these studies, reproducibility of performance is investigated over three trials. Yet, the third trial may not truly reflect a consistent performance, as novice participants have also displayed an increase in power output between successive trials when 3-km TTs are repeated six times (Foster et al., 2009). Taken together, these contrasting findings highlight the need for clear familiarization procedures (i.e., protocol and number of trials) for novice participants, to reduce systematic error (Hopkins, 2000). For experienced participants, at least one familiarization is recommended for reproducibility of performance (Laursen et al., 2003; Zavorsky et al., 2007; Abbiss et al., 2008; Stone et al., 2011). Additionally, at least one practice trial would be beneficial for experienced cyclists to develop a stable pacing strategy (Thomas et al., 2012). Yet, for novice participants, there are conflicting reports on the minimum number of familiarization trials before a pacing profile can be reproduced (Corbett et al., 2009; Foster et al., 2009).

Furthermore, it is not clear what protocol a familiarization trial should consist of, and there is a lack of data on such aspects. For an exercise that employs a similar intensity, it may be possible to use a different familiarization duration, as this experience can lead to the development of mental representations for the exercise to be performed (Micklewright et al., 2010). For example, when conducting 4- and 6-km TTs in a random order, experienced cyclists can retain a pacing strategy that does not negatively impact performance. This is likely a result of similar TT distances (4- vs. 6-km) conjuring a previous pacing strategy that needs only minor adjustment (Mauger et al., 2010). It is yet to be established if a similar finding would occur in novice participants who have no extensive experience to recall from. Such a finding may be beneficial when familiarizing a participant to a long duration exercise (e.g., 20-km cycling TT), as a shorter duration familiarization may be just as efficient to generate a reproducible performance. In conjunction with having limited experience in the exercise, another factor to consider for novice participants is the familiarity with the testing equipment. For exercise tests that allow adjustments in pace, a poor understanding of the testing equipment may negatively influence overall performance and the development of a pacing strategy. However, to the author's knowledge, it has yet to be investigated how testing equipment familiarity alone may influence performance. In fact, there are currently no clear guidelines for familiarizing novice participants to a cycling TT. As such, it is unclear if findings from previous research using a TT to measure an intervention are due to the intervention or simply a reflection of a variable pacing strategy.

The aims of this study were to investigate how performance is influenced by the duration and type of a familiarization protocol, and to establish the number of familiarization trials required to develop a stable pacing profile over multiple trials. It was hypothesized that it would take more than one practice for novice participants to establish a stable pacing profile, and a similar, but not identical, exercise may also provide a sufficient familiarization to a maximal physical task.

## Methods

### Participants

Thirty (18 female and 12 male) participants who were recreationally active, whilst relatively inexperienced at cycling, volunteered for this study (Table 1). It was required that participants did not have an extensive cycling history, were not currently active in cycling and had never previously completed a cycling TT. Participants were asked to refrain from any physical activity causing severe fatigue in the 36 h prior as well as any caffeine intake 2 h prior to testing. Prior to commencing the study, all participants were screened for risk factors and suitability to the exercise using a medical questionnaire. This study was carried out in accordance with the recommendations of the National Statement on Ethical Conduct in Human Research as described by the National Health and Medical Research Council (NHMRC) of Australia. All experimental testing was conducted with the prior approval from Victoria University's Human Research Ethics Committee. All participants gave written informed consent in accordance with the Declaration of Helsinki.

**Table 1:** Group anthropometric data.

**Measure**	**FF (*n* = 10)**	**HF (*n* = 10)**	**EF (*n* = 10)**	***P*-value**
Age (years)	21.40 ± 1.27	24.40 ± 6.36	23.40 ± 6.40	0.72
Height (cm)	169.75 ± 6.98	168.80 ± 7.41	173.70 ± 7.14	0.24
Body mass (kg)	68.74 ± 5.60	67.12 ± 13.90	67.22 ± 7.72	0.94
PPO (W)	286.80 ± 27.10	269.80 ± 37.58	289.10 ± 51.34	0.47
PPO (W/kg)	4.17 ± 0.37	4.10 ± 0.52	4.35 ± 0.86	0.78
VO_2peak_ (ml.min.kg^−1^)	42.40 ± 3.37	40.60 ± 5.25	43.40 ± 9.19	0.56
VO_2peak_ (L.min^−1^)	2.91 ± 0.32	2.69 ± 0.50	2.90 ± 0.64	0.60

*Data presented as mean ± SD. Each group n = 10, which consists of n = 6 females and n = 4 males. PPO, peak power output obtained from the incremental test. VO_2peak_, peak oxygen consumption. FF, full familiarization; HF, half familiarization; EF, equipment familiarization*.

### Experimental procedures

Participants were required to attend seven sessions, which involved one familiarization session, five self-paced 20-km cycling TT sessions, and one post-testing maximal incremental test for assessment of cardiorespiratory fitness. To ensure the experimental protocol was novel to participants, a 20-km cycling TT was utilized as it was expected that even with limited cycling experience, this exercise would be unknown to participants. Upon recruitment, a selective random process ensuring gender balance was used to assign participants to one of three familiarizations groups: *full* (FF), *half* (HF), or *equipment* (EF) familiarizations. In FF, participants performed a 20-km TT; in HF, they performed a 10-km TT, whilst in EF they performed 5 min of constant pace (75 W) cycling, which enabled participants to learn the mechanics of the bike, without experiencing a self-paced TT (Table 2). EF was included in the study design as a control group to quantify the variability of performance based on having no experience in the experimental exercise, but some familiarity with the testing equipment. To limit the influence of other external factors, all testing sessions were conducted at the same location and time of day (~1 h), separated by a minimum of 48 h.

**Table 2:** Overall performance data for the familiarization session.

**Measure**	**FF**	**HF**	**EF**
Duration (s)	2708.35 ± 404.10	1362.77 ± 133.36	299.99 ± 0.01
Mean power (W)	122.71 ± 33.22	118.59 ± 30.51	75.00 ± 0.00
Mean power (% of PPO)	43.20 ± 11.82	43.91 ± 8.57	26.63 ± 4.34

*Data presented as mean ± SD. TT, time-trial; FF, full familiarization; HF, half familiarization; EF, equipment familiarization*.

### Time-trials

All exercise was conducted on a Velotron Pro cycle ergometer (RacerMate Inc., Seattle, WA, USA). Prior to each testing session, a factory calibration was performed using the Accuwatt “run down” verification program (RacerMate Inc.) accompanying the ergometer software. Within the familiarization, participants set the ergometer to their own specifications, with values recorded and replicated for subsequent sessions. All TT protocols were controlled via Velotron Coaching software (Version 1.6.458, RacerMate Inc.) with all courses being flat with no wind effect. TTs were conducted in the same laboratory, with regulated environmental conditions (Temperature 22.4 ± 1.1°C, humidity 50.8 ± 7.5%, and barometric pressure 762.9 ± 4.6 mmHg). No fan was provided to participants in all TTs, although they were permitted to drink water *ad libitum*. Participants were asked to remain seated throughout the entire protocol, and toe clips were used to prevent feet from slipping.

Preceding the TT, a warm-up (5-min cycling at 75 Watts) was conducted. In all TTs, participants were instructed to finish the required distance “as quickly as possible” by being free to change gear and cadence throughout the trial to what felt appropriate at the time. Changing of gear utilized the ergometer electronic gearing system with all TTs started in the same gear. To overcome flywheel inertia, participants were instructed to obtain a self-selected comfortable cadence immediately prior to beginning the trial, with the TT commencing with a verbal 3-s countdown from the researcher. Throughout all TTs, participants were blinded from all performance information, except for distance covered, and received no encouragement from investigators. Participants did not receive any information on how they performed until all TTs were completed (Sporer and McKenzie, 2007).

### Maximal incremental test

After all TTs were completed, a maximal incremental test was conducted to characterize participant's peak oxygen uptake (VO_2peak_) and peak power output (PPO). The maximal incremental test involved incremental stages of 30 W/min commencing after a 3-min baseline period, cycling at a 30 W (females), or 60 W (males) resistance which is like the protocols used for those unaccustomed to cycling tasks (Williams et al., 2012). Participants were encouraged throughout the final stages and the test ceased when the participant could not maintain a cadence above 60 rpm or volitional fatigue was achieved.

Prior to the incremental test O_2_ and CO_2_ gas was calibrated with known concentrations and flow calibrations were performed using a 3-L calibration syringe. Participants were fitted with a headpiece to assist the appropriate function of a Hans-Rudolph two-way non-rebreathing valve. Expired gas was collected and analyzed every 15-s (S-3A/I (O_2_) and CD-3A (CO_2_), AEI Technologies Inc., Pittsburgh, PA). VO_2_peak was calculated as the highest 30-s mean VO2. Peak power was extrapolated by using the formula [Peak power last completed stage (Watts) + time in the last stage (s)/60 × 30 (Watts)] (Lima-Silva et al., 2013).

### Statistical analysis

All data was analyzed using SPSS (version 22, SPSS Inc., Chicago, IL) with data reported as mean ± *SD*. All data was tested for normality (Shapiro–Wilk test). When normality assumptions were violated an equivalent non-parametric test was performed. Tests for homogeneity of variances were performed to ensure normality of the cohort for dependent variables. When homogeneity of variances was violated Welch *F*-ratio is reported. The level of significance for all tests was set at *P* < 0.05. In the instance of a significant main or interaction effect, *post-hoc* Sidak comparisons and *t*-tests were conducted to examine differences and its magnitude (effect size). Effect sizes for one-way and repeated-measures ANOVAs are reported as partial eta squared (η_*p*_^2^) with a small effect at 0.01–0.059, medium effect 0.06–0.139 and a large effect > 0.14. Effect sizes for *t*-tests are reported as Cohen's *d* with a small effect being 0.2–0.49, medium 0.5–0.79 and large > 0.8 (Cohen, 1988).

For data analysis, given the inter-participant differences in TT power output, power has been reported as a percentage of the individual's PPO obtained from the maximal incremental (i.e., % of PPO). To examine differences between participants in the three different groups (FF, HF, and EF), one-way ANOVAs were conducted on anthropometric variables.

#### Analysis of between and within groups TT variability

To explore whether the three different familiarization protocols had an influence on performance in terms of TT duration and TT mean power, we conducted a three (FF, HF, and EF) by five (TT) repeated-measure ANOVA. Follow-up one-way ANOVAs were conducted to investigate differences between familiarization protocols (*n* = 3), with follow-up *t*-tests conducted to determine the effect size between familiarization protocols for each TT. To examine differences between trials within-groups, one-way repeated-measures ANOVAs (trials, *n* = 5) were conducted, with follow-up *t*-tests conducted to determine the effect size between TTs for each familiarization protocol. In addition, as the FF group familiarization was the same protocol as the TTs, *t*-tests were conducted to determine the difference between the familiarization trial and all other TTs in the FF group.

To examine variability of performance over the five trials and between the three groups (FF, HF, and EF), we calculated the coefficient of variation (CV) using the formula $CV_{1,2}=SD_{1,2}/{\overset{-}{x}}_{1,2}\times 100$. CV was calculated for TT duration and TT mean power with individual CV's calculated and averaged for groups.

#### Analysis of pacing profiles

To prepare for pacing profile analysis, mean power output profiles were established by normalizing each TT to 2,000 data points (Smits et al., 2016). To examine the development of pacing profiles within each group, mean power output profiles were analyzed by applying a regression model to establish the line of best-fit profile. The line of best-fit was established by considering the regression model with the highest explained variance (*R*^2^) for each mean power output profile. To compare the within-groups between trials variance, we considered the magnitude of change in *R*^2^ between trials as an indicator of variability in pacing profiles, with smaller changes in *R*^2^ considered as lower variability between trials.

## Results

There were no between-group differences for any anthropometric variables (Table 1).

### Analysis of between and within groups TT variability

**Figure 1:**
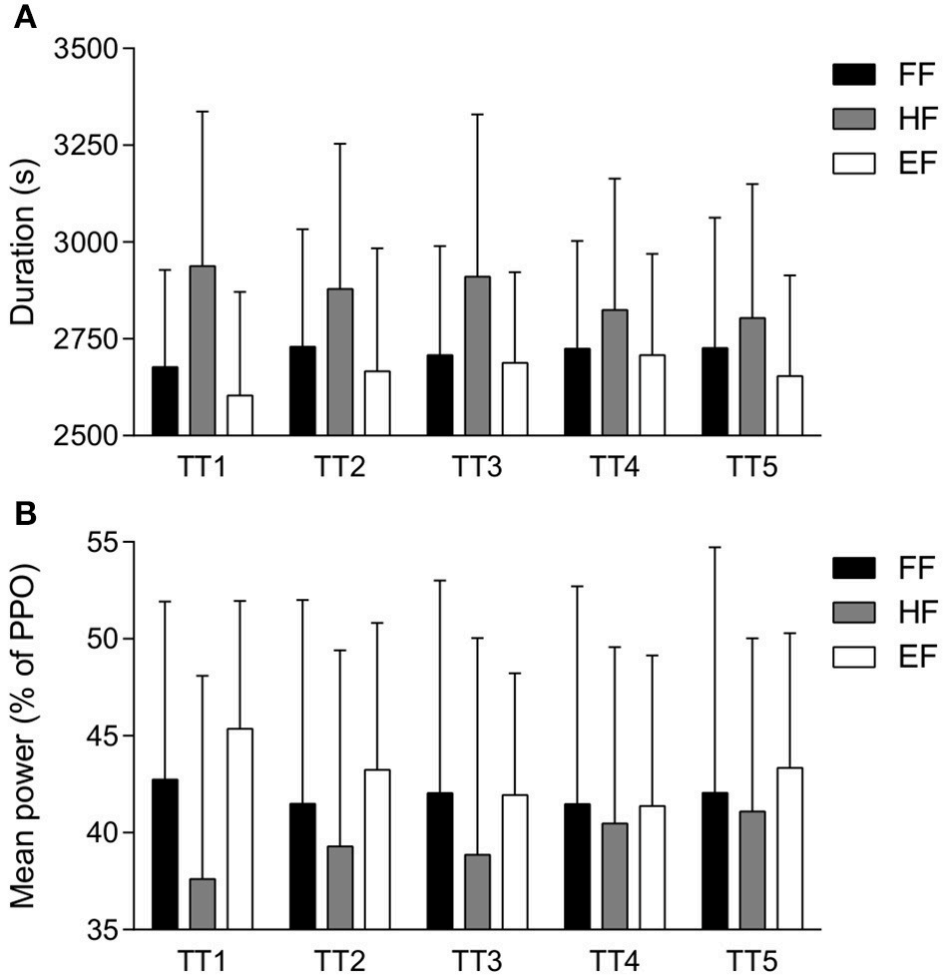
Mean ± *SD* TT performance measures. TT duration (s) **(A)** and mean power output (% of PPO) **(B)** for each TT. TT, time-trial; FF, full familiarization; HF, half familiarization; EF, equipment familiarization.

A significant interaction effect (protocol × trial) with large effect sizes was found for TT duration (*P* = 0.02, η_*p*_^2^ = 0.16) and mean power (*P* = 0.02, η_*p*_^2^ = 0.15; Figure 1). Sidak *post-hoc* comparisons exploring differences between the three groups at each trial did not show any significant differences. Visual inspection indicates that the biggest difference between groups was in TT1, with HF group having an increased TT duration (Figure 1A) and decreased mean power (Figure 1B) compared to FF and EF.

One-way repeated-measures ANOVAs to locate differences between trials within each group showed no significant differences for TT duration (Table 3). There was a significant effect for mean power in HF and EF groups but not for FF. *Post-hoc* comparisons did not reveal any differences. For both TT duration and mean power in both HF and EF, the effect size (η_*p*_^2^) was found to be large (Table 3). Between trials, the effect size (*d*) was smaller in the FF group compared to HF and EF. For FF group, there were small effect sizes between the familiarization and TT1, whilst between TT1 and TT2, the effect sizes were moderate. For HF and EF there were large effect sizes between TT1 and the other trials (Table 3). Reflecting effect size differences, CV data between successive trials is provided in Table 4. Overall, the lowest CV for TT duration and mean power occurred in FF group between TT3 and TT4, with TT4-TT5 CV comparable but minimally increased.

**Table 3:** Within-group between TT differences in overall performance, with repeated measures ANOVA comparison and Cohen's *d* effect size for between TT differences.

**Duration (s)**							**Mean power (% of PPO)**						
**Group**	**Trial**	**TT1**	**TT2**	**TT3**	**TT4**	**TT5**	**Group**	**Trial**	**TT1**	**TT2**	**TT3**	**TT4**	**TT5**
**FF**							**FF**						
	*FAM*	31.01 (0.19)	−20.99 (−0.23)	0.56 (0.00)	−16.01 (−0.10)	−18.17 (−0.11)		*FAM*	0.45 (0.13)	1.70 (0.73)	1.16 (0.34)	1.72 (0.43)	1.14 (0.27)
**FF**							**FF**						
*P* = 0.69 $\eta_{p}^{2}$ = 0.05	*TT1*	–	−52.00 (−0.55)	−30.46 (−0.55)	−47.03 (−0.65)	−49.19 (−0.70)	*P* = 0.79 $\eta_{p}^{2}$ = 0.03	*TT1*	–	1.25 (0.52)	0.71 (0.34)	1.27 (0.46)	0.69 (0.24)
	*TT2*		–	21.55 (0.33)	4.97 (0.06)	2.81 (0.03)		*TT2*		–	−0.54 (−0.34)	0.02 (0.01)	−0.56 (−0.24)
	*TT3*			–	−16.57 (−0.49)	−18.73 (−0.49)		*TT3*			–	0.55 (0.53)	−0.02 (−0.02)
	*TT4*				–	−2.16 (−0.04)		*TT4*				–	−0.58 (−0.40)
**HF**							**HF**						
*P* = 0.08 $\eta_{p}^{2}$ = 0.26	*TT1*	–	59.02 (1.36)	27.33 (0.65)	113.85 (1.84)	134.41 (1.54)	*P* = 0.03 $\eta_{p}^{2}$ = 0.25	*TT1*	–	−1.68 (−1.06)	−1.25 (−1.07)	−2.86 (−1.82)	−3.48 (−1.62)
	*TT2*		–	−31.69 (−0.50)	54.83 (1.53)	75.39 (1.01)		*TT2*		–	0.43 (0.25)	−1.18 (−1.32)	−1.80 (−0.97)
	*TT3*			–	86.52 (1.03)	107.08 (1.02)		*TT3*			–	−1.61 (−0.93)	−2.23 (−0.95)
	*TT4*				–	20.56 (0.41)		*TT4*				–	−0.62 (−0.49)
**EF**							**EF**						
*P* = 0.07 $\eta_{p}^{2}$ = 0.21	*TT1*	–	−62.24 (−1.41)	−84.09 (−1.36)	−104.52 (−1.82)	−50.28 (−1.16)	*P* = 0.04 $\eta_{p}^{2}$ = 0.24	*TT1*	–	2.12 (1.37)	3.41 (1.47)	3.97 (1.91)	2.02 (1.15)
	*TT2*		–	−21.86 (−0.36)	−42.29 (−0.71)	11.95 (0.19)		*TT2*		–	1.29 (0.61)	1.86 (0.94)	−0.10 (−0.05)
	*TT3*			–	−20.43 (−0.39)	33.81 (0.70)		*TT3*			–	0.57 (0.29)	−1.39 (−0.80)
	*TT4*				–	54.24 (1.12)		*TT4*				–	−1.96 (−1.22)

*Data presented as the mean difference between TTs and (ES). Effect sizes in parentheses are presented as Cohen's d for differences in paired sample t-tests between trials. Values for Cohen's d are small effect at 0.2–0.49, medium 0.5–0.79 and large > 0.8. TT, time-trial; FAM, familiarization; $\eta_{p}^{2}$, partial eta squared; FF, full familiarization; HF, half familiarization. EF, equipment familiarization*.

**Table 4:** Within-group CV between trials.

	**Duration (s)**			**Mean power (% of PPO)**		
	**FF**	**HF**	**EF**	**FF**	**HF**	**EF**
CV FAM-TT1	4.67			10.78		
CV TT1-TT2	3.42	2.28	2.02	8.06	5.55	4.90
CV TT2-TT3	2.18	2.31	2.95	4.99	5.07	6.80
CV TT3-TT4	1.47	3.09	2.21	3.42	6.76	5.45
CV TT4-TT5	1.89	1.79	2.40	4.26	4.06	5.94

*Data presented as mean CV. CV, coefficient of variation; TT, time-trial; FAM, familiarization; FF, full familiarization; HF, half familiarization; EF, equipment familiarization*.

One-way ANOVAs between groups revealed no significant difference for TT duration and mean power in any TT (Table 5). However, there was a large effect size (η_*p*_^2^) for differences in TT1 duration and a moderate effect size for TT1 mean power. Between HF and EF groups, there was a large effect size (*d*) for TT1 duration and mean power. Between HF and FF groups, there was a large effect size (*d*) for TT1 duration, whilst mean power effect size (*d*) was moderate. The comparisons between FF and EF had a small effect size (*d*), whilst moderate effect sizes (*d*) were observed in TT2, TT3, TT5 duration between HF and EF groups (Table 5).

**Table 5:** Between-group differences in overall performance, with one-way ANOVA comparison for each trial and Cohen's *d* effect size for between-group differences.

**Duration (s)**				**Mean power (% of PPO)**			
**Trial**	**Group**	**HF**	**EF**	**Trial**	**Group**	**HF**	**EF**
**TT1**				**TT1**			
*P* = 0.06 $\eta_{p}^{2}$ = 0.19	*FF*	−260.76 (−0.83)	73.71 (0.30)	*P* = 0.16 $\eta_{p}^{2}$ = 0.13	*FF*	5.14 (0.55)	−2.61 (−0.34)
	*HF*	−	334.47 (1.04)		*HF*	−	−7.74 (−0.93)
**TT2**				**TT2**			
*P* = 0.36 $\eta_{p}^{2}$ = 0.07	*FF*	−149.75 (−0.46)	63.48 (0.22)	*P* = 0.65 $\eta_{p}^{2}$ = 0.03	*FF*	2.20 (0.23)	−1.74 (−0.20)
	*HF*	−	213.22 (0.65)		*HF*	−	−3.94 (−0.46)
**TT3**				**TT3**			
*P* = 0.25 $\eta_{p}^{2}$ = 0.10	*FF*	−202.98 (−0.60)	20.07 (0.08)	*P* = 0.71 $\eta_{p}^{2}$ = 0.02	*FF*	3.17 (0.30)	0.09 (0.01)
	*HF*	−	223.05 (0.69)		*HF*	−	−3.09 (−0.36)
**TT4**				**TT4**			
*P* = 0.64 $\eta_{p}^{2}$ = 0.03	*FF*	−99.89 (−0.34)	16.22 (0.06)	*P* = 0.97 $\eta_{p}^{2}$ < 0.01	*FF*	1.01 (0.10)	0.10 (0.01)
	*HF*	−	116.11 (0.40)		*HF*	−	−0.91 (−0.11)
**TT5**				**TT5**			
*P* = 0.58 $\eta_{p}^{2}$ = 0.04	*FF*	−77.17 (−0.24)	72.61 (0.25)	*P* = 0.88 $\eta_{p}^{2}$ = 0.01	*FF*	0.97 (0.09)	−1.28 (−0.13)
	*HF*	−	149.78 (0.52)		*HF*	−	−2.25 (−0.30)

*Data presented as the mean difference between TTs and (ES). Effect sizes in parentheses are presented as Cohen's d effect size for differences in independent sample t−tests between groups for each trial. Values for Cohen's d are small effect at 0.2–0.49, medium 0.5–0.79, and large >0.8. TT, time−trial; $\eta_{p}^{2}$, partial eta squared; FF, full familiarization; HF, half familiarization; EF, equipment familiarization*.

### Analysis of pacing profiles

**Figure 2:**
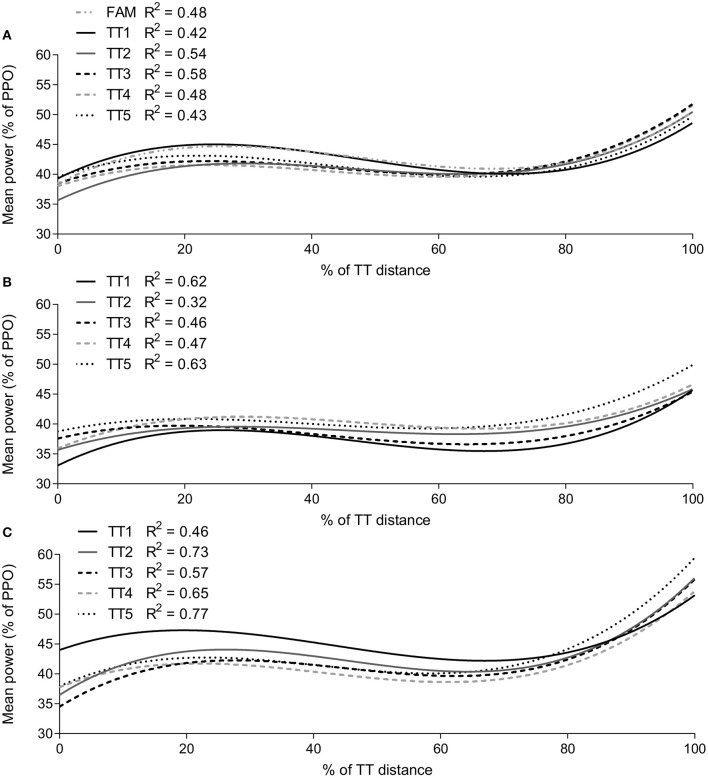
The line of best fit for each 2,000-point mean power output profile. FF **(A)**, HF **(B)**, EF **(C)**. TT, time-trial; FAM, familiarization; FF, full familiarization; HF, half familiarization; EF, equipment familiarization. *R*^2^, explained variance. FAM (dash and dot gray), TT1 (solid black), TT2 (solid gray), TT3 (dashed black), TT4 (dashed gray), TT5 (dotted black).

The line of best-fit characteristics for mean power output profiles are shown in Figure 2. The highest explained variance (*R*^2^) for all trials was established with the application of a cubic regression model. Change in *R*^2^ between the five trials was lowest in FF group, ranging from 0.43 to 0.58 (Figure 2A), HF group ranged from 0.32 to 0.63 (Figure 2B) and EF group ranged from 0.46 to 0.77 (Figure 2C).

## Discussion

This study investigated the efficacy of differing familiarization protocols to limit the variability of pacing and performance over multiple trials. The main finding is that a variability in pacing and performance was lowest after a full familiarization and two additional TTs, while four or more trials did not improve results. This indicates that multiple familiarizations of a self-paced exercise are required before experimental testing to achieve enhanced reproducibility.

### Variability in performance

Previous exercise experience provides relevant information to determine an appropriate pacing strategy for subsequent trials that will lead to optimal performance (Ulmer, 1996; Tucker, 2009). Therefore, with different levels of experience (i.e., familiarization), it is not surprising to observe large effects for between group performance differences occurring in TT1 in novice participants (Table 5 and Figure 1). For TT1, the difference between FF and EF group was small (Table 5), however, there were large effects in comparison to HF group. This suggests a familiarization that is not identical to the exercise (i.e., HF) is less effective than very limited experience (i.e., EF) for best performance in one trial. This contrasts with anticipatory regulation models, as no experience would presumably create a poor understanding of exercise demands (Tucker, 2009), yet, it appears for novice participants, the experience of a similar mean power output sustained for half the duration (i.e., HF) is detrimental to performance. In comparison to no experience, this similar experience likely creates a discrepancy between the perceived and actual demands of the exercise, with a substantial change to the pacing strategy required. Similar changes in pacing have been demonstrated when exercise distance is varied, either knowingly (Billaut et al., 2011) or as a deception (Paterson and Marino, 2004). As TTs were repeated, pacing in all groups presumably becomes more refined and differences between groups were gradually reduced so that performance was comparable in TT4 and TT5 (Table 5).

Within HF and EF groups, but not FF, there were large effect sizes (η_*p*_^2^) for changes across all five TTs for performance measures (Table 3). This suggests that application of one FF is a superior familiarization protocol for reproducibility of performance over multiple trials. In addition, when comparing performance differences after completing one 20-km TT (i.e., the difference between familiarization and TT1 in FF, and the difference between TT1 and TT2 in HF and EF), there were small differences in FF group, but large effects in HF and EF (Table 3). This finding suggests that HF and EF may be detrimental to performance over several trials, and one full familiarization may be adequate for reproducibility of performance. Yet, as there was a moderate effect size (*d*) between TT1 and all other TTs in FF group (Figure 1 and Table 3) it appears that it may be prudent to implement more than one familiarization. Well-trained cyclists need only one familiarization trial to stabilize performance in a 20-km TT (Zavorsky et al., 2007), and this finding is also true for less experienced participants conducting 2-km TTs (Corbett et al., 2009). A 2-km TT, however, elicits a different pacing strategy than a 20-km TT (Abbiss and Laursen, 2008). In addition, the mechanisms of fatigue are likely to differ within shorter tasks, thus requiring less regulation of intensity (Tucker, 2009), presumably making it easier for untrained participants to pace appropriately. This current investigation was designed to confront participants with an exercise that is relatively novel and highly dependent on complex internal regulation (Tucker, 2009). Therefore, it was expected that findings would give a better indication of the number of trials needed before reproducibility is obtained in novice participants. In support of our finding, trained cyclists have exhibited continued improvement after a second 8-mile TT (Noreen et al., 2010). However, as recent experience was lacking in these participants, it is possible that they needed to be re-familiarized, and the use of a pacer may have also influenced motivation. Regardless, this provides support for multiple familiarizations to reduce systematic error over multiple trials.

To indicate the number of trials required before variability in performance is reduced, performance measures were compared between trials (Table 3). Within FF Group, excluding the comparison with TT1 and mean power between TT3-TT4, the effect size (*d*) for performance changes between later TTs was small (Table 3). In contrast, there were large effects between later TTs for both HF and EF groups (Table 3). In conjunction, the between trial CV for TTs was lowest in FF group (Table 4), with CV for TT duration between TT3-TT4 and TT4-TT5 comparable to reported values from competitive cyclists (Zavorsky et al., 2007; Noreen et al., 2010). This strongly suggests that three full familiarizations can adequately reduce the variability in novice participants to comparable levels of experienced individuals. This finding is particularly important for researchers aiming to determine the magnitude of an intervention in novice participants. Although overall performance can be similar, the way in which the exercise is paced may differ (Ansley et al., 2004). In this regard, an understanding on how TTs were paced would give important information on reproducibility of performance and whether a pacing profile has been established.

### Variability in pacing profiles

Previous experience allows for an understanding of the physiological demands of the exercise so that an appropriate intensity can be initially set that requires less refinement throughout the task (Mauger et al., 2009; Tucker, 2009; Micklewright et al., 2010). This has been demonstrated by participants with minimal experience increasing their starting power output when both 3- and 10-km cycling TTs were repeated (Foster et al., 2009). Furthermore, without a familiarization, it was only by the third or fourth TT that a similar pacing profile was achieved (Foster et al., 2009). In our investigation, the variability of pacing profiles (i.e., the magnitude of change in *R*^2^ between trials) was lowest within FF group (Figure 2A). Although the pacing profile produced in the familiarization and TT1 was similar, there is an apparent difference of TT2 in FF group (Figure 2A). Most importantly, and in agreement with previous research, a visual representation of power output shows a similar pacing profile in TT3 and later TTs (Figure 2A). This observation further strengthens the argument for multiple trials to familiarize novice participants. It should be noted, that the pacing profiles observed in this study will likely differ to a trained athlete (Foster et al., 2009) presumably due to participant capabilities and the willingness to exercise maximally (Edwards and Polman, 2013). However, the aim of this investigation was to observe how variability changed between TTs so a baseline performance could be identified. In this regard, these results provide evidence that three familiarizations should be administered to establish a reliable baseline in novice participants and to enhance reproducibility over several trials.

In all groups, there was an apparent difference in the pacing profile of TT1 (Figure 2), with EF TT1 the most dissimilar to other TTs (Figure 2C). The higher intensity at the start of the TT1 is surprising given the lack of experience in the exercise, as it would be expected a more conservative approach would be taken (i.e., an intensity that can be maintainable for an extended period; Lambert et al., 2005; Tucker, 2009; Williams et al., 2012). No experience of the exercise may have created a discrepancy between the perceived and actual demands of the exercise. Subsequently, the fast start strategy in TT1 may have contributed to reduced reproducibility of the pacing profile among the five TTs (Figure 2C). In contrast, a conservative approach was taken by HF participants, with much of TT1 having a reduced power output compared to later TTs (Figure 2B). Much like what was observed in EF group, this difference likely occurs as the difference in experience creates a poor understanding of actual exercise demands. It is possible that participants were conservative during early TTs as they anticipated a greater metabolic cost than what was experienced in the familiarization. However, as experience in the TT was gained, the intensity was increased in later TTs (Figures 1B, 2B), as it becomes apparent that the TT could be completed without negative consequences. This willingness to work at a higher intensity as participants become more experienced in the exercise has been demonstrated previously (Foster et al., 2009).

Another indication of pacing variability is that the end spurt of TTs became larger as TTs were repeated in both HF and EF groups. This likely indicates the adoption of a different pacing strategy in the early part of TTs. Specifically, with more experience, participants have a greater understanding of exercise demands and presumably adopt a more manageable approach during the TT that allows a higher intensity toward the end of the bout (Micklewright et al., 2010; Williams et al., 2012). Along with this line of reasoning, participants in EF had a reduced end spurt in TT1 with the early unsustainable intensity in the first half of TT1 likely creating greater fatigue (Figure 2C), thus diminishing the ability to work at a higher intensity at the end of the TT. In contrast to both HF and EF groups, there is no substantial change in the end spurt between TTs in the FF group. It is most likely the full prior experience from a familiarization allowed participants to gain a better understanding of the exercise and may have adopted a more efficient even paced strategy (Abbiss and Laursen, 2008).

### Limitations

In conjunction with the influence of prior experience, the knowledge of the actual performance of a task is also crucial in setting a successful pacing strategy (Micklewright et al., 2010). Information was withheld until completion of our study and therefore can be considered a limitation, as our participants may have reduced their variability between TTs if they were made aware of their performances. However, this information would have introduced a bias to our objective, so we preferred to withhold this information.

### Practical applications

This investigation refines guidelines for familiarizing novice participants to a cycling TT. Application of a full exercise familiarization produced the greatest reproducibility in pacing and performance over multiple trials, compared to a half exercise familiarization and no experience. Within the FF group, there were small differences in TT duration between TT2 and other TTs, indicating only two familiarizations may be required. Yet, a stable pacing profile was not developed until TT3 (Figure 2A). Taken together, these results provide support for the use of three full exercise familiarizations to minimize the variability often demonstrated by novice participants. In addition, the cohort of participants in this study had no experience in self-paced TTs and had limited experience in cycling. Taking this into account, it may be possible to extend the conclusion that three familiarizations should be administered to reduce variability in any self-paced exercise. It has also been observed that trained cyclists can display learning effects after a second TT (Noreen et al., 2010). In this situation, recent experience in the exercise was lacking, suggesting experienced participants may need to be re-familiarized to the TT. With novice participants, as a worst-case scenario, this study gives merit to the use of multiple familiarizations to reduce systematic error, regardless of participant experience. However, it would likely warrant future investigations to determine if the same effects are seen in different modes of exercise, and the magnitude of effect in trained participants who lack specific experience in the exercise. Transferring our recommendations into practice, the application of three familiarizations may considerably add to lengthy testing protocols and likely impact on participant recruitment and retention. To address this, it may be possible to reduce the time interval between familiarization sessions without compromising pacing strategy development. In this study, trials were conducted at least 48 h apart, however, for shorter TTs, similar to the protocol utilized by Mauger et al. (2009), it may be possible to conduct multiple trials within one session, to achieve a reproducible performance. Although this would require further investigation in a novice cohort.

## Conclusions

Prior experience is an important moderator of self-paced performance. Therefore, it is important for participants to gain experience in an exercise before conducting experimental testing to establish a reproducible baseline performance. This study demonstrates that three familiarization trials of the exact experimental protocol should be administered to reduce variability across multiple trials in novice participants. This finding should be considered when interpreting the results of interventions that utilize self-paced tasks and unfamiliar participants. In conclusion, it is recommended that future investigations administer three familiarization trials to reduce systematic error before experimental testing.

## Author contributions

Conceived and designed the experiments: AH, FB, MV, and RP. Collected and analyzed data: AH. Interpreted results of research: AH, FB, and RP. Drafted manuscript and prepared table/figures: AH. Edited, critically revised manuscript and approved the final version of manuscript: AH, FB, MV, and RP.

### Conflict of interest statement

The authors declare that the research was conducted in the absence of any commercial or financial relationships that could be construed as a potential conflict of interest.
